# Interference among the Processing of Facial Emotion, Face Race, and Face Gender

**DOI:** 10.3389/fpsyg.2016.01700

**Published:** 2016-10-28

**Authors:** Yongna Li, Chi-Shing Tse

**Affiliations:** ^1^Department of Psychology, Renmin University of ChinaBeijing, China; ^2^Department of Educational Psychology, Chinese University of Hong KongHong Kong, Hong Kong; ^3^Centre for Learning Sciences and Technologies, Chinese University of Hong KongHong Kong, Hong Kong

**Keywords:** facial emotion, gender, race, face processing, face perception

## Abstract

People can process multiple dimensions of facial properties simultaneously. Facial processing models are based on the processing of facial properties. The current study examined the processing of facial emotion, face race, and face gender using categorization tasks. The same set of Chinese, White and Black faces, each posing a neutral, happy or angry expression, was used in three experiments. Facial emotion interacted with face race in all the tasks. The interaction of face race and face gender was found in the race and gender categorization tasks, whereas the interaction of facial emotion and face gender was significant in the emotion and gender categorization tasks. These results provided evidence for a symmetric interaction between variant facial properties (emotion) and invariant facial properties (race and gender).

## Introduction

Human faces convey significant amounts of information during social interaction. People with expertise in face processing can quickly and simultaneously process facial information from multiple dimensions. Previous studies focused on the concurrent processing of two dimensions, such as race and emotion (e.g., Hugenberg, 2005; Craig et al., 2012), race and gender (e.g., Zhao and Hayward, 2013; Carpinella et al., 2015), and emotion and gender (e.g., Karnadewi and Lipp, 2011). These studies examined the interaction between dimensions on face recognition and whether they were symmetric or asymmetric. ‘Symmetric interaction’ indicates that dimension X affects the processing of dimension Y and dimension Y also affects the processing of dimension X. For example, when categorizing racially ambiguous faces, White participants were faster to categorize the target face as White than as Black/Asian for female faces and were faster to categorize the target face as Black/Asian than as White for male faces. That is, race categorization is biased by face gender (Carpinella et al., 2015). Other works revealed more errors in gender categorization for Black women than for Black men, White women, and White men (Goff et al., 2008). Face race and face gender, which are invariant face properties, are processed in an integrative way, such that the analysis of one attribute is affected by automatic processing of the other (Zhao and Hayward, 2013). ‘Asymmetric interaction’ occurs when dimension X affects the processing of dimension Y, but dimension Y does not affect the processing of dimension X. For example, Karnadewi and Lipp (2011) used the Garner paradigm that consisted of an orthogonal and a control condition to examine the processing of invariant and variant face properties. The Garner paradigm was originally developed to investigate whether two factors (e.g., object shape and color) were processed independently or interactively (Garner, 1974). Both task-relevant and task-irrelevant factors vary in the orthogonal condition and only the task-relevant factor varies in the control condition. Karnadewi and Lipp (2011) required people to perform race and emotion, gender and emotion, and age and emotion categorization tasks. Their participants responded slower in the orthogonal condition than in the control condition in emotion categorization but not in race, gender, or age categorization. Hence, they concluded that face race, gender, and age affected emotion categorization but facial emotion had no effect on race, gender, or age categorization. Nevertheless, to our knowledge no study has simultaneously manipulated three dimensions of facial cues (facial emotion, race, and gender) and tested their symmetric or asymmetric interactive influence on face processing in emotion, race, and gender categorization tasks. In three experiments with faces of three races as stimuli, the current study aimed at filling this gap in the literature.

### Facial Emotion and Face Race Interaction

Automatically perceived race cues modulated the processing of emotion in a facial emotion categorization task. For example, White participants classified happiness faster than anger/sadness on same-race (White) faces, but they were slower to do so on other-race (Black) faces (Hugenberg, 2005). White participants detected an angry expression more quickly on Black faces than White faces, and estimated that an angry expression lasted longer on Black faces than on White faces (Hugenberg and Bodenhausen, 2003). Facial emotion and face race interacted in fear conditioning (Bramwell et al., 2014). Zebrowitz et al. (2010) reported that face race affected neutral face resemblance to emotional expressions. For White participants, White neutral facial expressions objectively resembled White angry facial expressions, but this did not occur for Black or Korean faces. Participants were more able to identify emotion expressed on their own-race faces than other-race faces (Elfenbein and Ambady, 2002a,b; Elfenbein et al., 2002). These findings suggest that there is an ‘in-group’ advantage in emotion categorization. Subsequent studies showed that the type of facial emotion modulated this in-group advantage in emotion categorization. For example, Ackerman et al. (2006) reported that White participants more accurately identified neutral White faces than neutral Black faces, but were less accurate for angry White faces than for angry Black faces.

In contrast, Kubota and Ito (2007) used White and Black faces with happy, angry, and neutral expressions, and found an independent processing of face race and facial emotion for White participants in an emotion categorization task. Craig et al. (2012) argued that stimulus presentation duration (unlimited versus limited), stimulus type (computer-generated faces versus photos of real faces), and/or stimulus set size (small versus large) all modulated the face race x facial emotion interaction effect. Kubota and Ito (2007) also failed to find any effect of facial emotion on ‘own-versus-other’ race categorization (where race was task-relevant). Hence, the occurrence of facial emotion × face race interaction may depend on the task type.

### Facial Emotion and Face Gender Interaction

Studies on facial emotion × face gender interaction also revealed mixed findings. Becker et al. (2007) found that anger was better identified in male faces than in female faces, whereas happiness was better identified in female faces than in male faces. Face gender could interfere with emotion categorization, but facial emotion did not interfere with gender categorization (Plant et al., 2000; Atkinson et al., 2005). In contrast, Valdes-Conroy et al. (2014) found a facial emotion × face gender interaction in error rates in the gender categorization task and in the components of event-related potentials (N170) in the emotion categorization task. Whether the facial emotion × face gender interaction is symmetric or asymmetric is determined by stimulus set size. Larger sets of faces (e.g., 32 faces) produce a symmetric interaction, but smaller sets of faces (e.g., 4 faces) yield an asymmetric interaction (Lipp et al., 2015). However, Le Gal and Bruce (2002) failed to find any interaction between facial emotion and face gender, suggesting that these two factors could be independently processed in facial recognition.

The processing of facial emotion and face gender is also examined in paradigms other than face recognition. In a demanding matching task, fearful facial emotion is automatically processed and interferes with ongoing categorization decisions (Vuilleumier et al., 2001). Emotional faces (e.g., fearful, angry, or happy) capture individual’s attention in a visual search task, even as a task-irrelevant feature (Hodsoll et al., 2011). The event-relate potentials (ERPs) analyses showed that the processing of face gender occurred as early as 145–185 ms after the stimulus onset (Mouchetant-Rostaing et al., 2000). The automatic processing of facial emotion and face gender not only captures attention but also influences motoric action (Ambron and Foroni, 2015; Ambron et al., 2016).

### Face Race and Face Gender Interaction

Face race and face gender are two invariant face features that are processed automatically (Ito and Urland, 2003; Kubota and Ito, 2007) and analyzed by the same neural system (Haxby et al., 2000). Gender cues biased race categorization. When racially ambiguous faces became more feminine, White participants were more likely to classify them as White than to classify them as Black or Asian (Carpinella et al., 2015). There was also some evidence for race cues biasing gender categorization. For example, Johnson et al. (2012) demonstrated that White participants categorized Black male faces more efficiently than White and Asian male faces, but this pattern did not occur in female faces. In a gender categorization task, participants’ responses to other-race faces could be impaired by the discrimination of face gender (O’Toole et al., 1996). On the other hand, Zhao and Bentin (2008) found that participants’ race and face race did not influence gender categorization.

The inconsistencies in the findings of the abovementioned studies may be due to the specific experimental paradigms used. For example, Karnadewi and Lipp ’s (2011, Experiment 1) Garner Paradigm showed that face race, age, and gender influenced the categorization of emotion (variant feature), but emotion showed no effect on the categorization of face race, age, and gender (invariant features). They attributed this asymmetric interaction to the possibility that the categorizations of invariant facial cues are faster than those of variant cues, such that the slower processing of variant cues, such as facial emotion, may not affect the faster processing of invariant cues, such as face race and age. In contrast, when the task involves categorization based on variant cues (e.g., emotion categorization), the effect of face race and age, which are processed more quickly, may be observed.

Previous studies also differed in the inclusion of neutral expression. Some compared neutral with either positive or negative facial emotion (Ackerman et al., 2006; Kubota and Ito, 2007), whereas others include only faces with positive and negative emotions, such as happy and angry expressions, with no neutral emotion condition as a control (Karnadewi and Lipp, 2011). It is impossible to tease apart the effect of positive and negative emotions when neutral expression condition was not included. The positive and negative emotions could affect the processing of other facial cues to the same extent and in the same direction, resulting in non-significant effects. For example, both angry and happy faces were more likely to be perceived as directly looking at the observer than neutral faces (Ewbank et al., 2009). If a study on facial emotion and gaze direction judgment only involves angry and happy faces, it would unlikely reveal any effect of emotion on gaze direction. The inclusion of the neutral condition may help to clarify the potential effect of different emotions on face categorization.

Most of previous studies focus only on two facial properties, for instance pairings of face race and facial emotion, face race and face gender, facial emotion and eye gaze, or facial emotion and face gender. In these studies, the interactive processing of various facial cues were not consistent. In the current study, the interaction between invariant and variant facial cues was more systematically tested. Three face categorization experiments were used to investigate the processing of all three facial properties (race, gender, and facial emotion) with the same set of facial stimuli and procedures (i.e., the event sequence of each trial). Chinese, White, and Black faces making happy, angry and neutral expressions were selected from different facial emotion databases. The proportion of male to female faces was 1:1 in all experiments. Experiment 1 was a three-alternative-forced-choice (happy, sad, or neutral) emotion categorization task, with face race and face gender as the task-irrelevant factors. Experiment 2 was a three-alternative-forced-choice (Chinese, White, or Black) race categorization task, with facial emotion and face gender as the task-irrelevant factors. Experiment 3 used a binary-choice gender categorization task, with facial emotion and face race as the task-irrelevant factors.

Given previous findings that (a) invariant facial cues (face race and face gender) interfere with the processing of variant facial cues (facial emotion) but not vice versa (Karnadewi and Lipp, 2011) and (b) multiple facial properties are processed simultaneously, we predicted a facial emotion × face race interaction, a facial emotion × face gender interaction, and a facial emotion × face race × face gender interaction in Experiment 1. That is, gender and/or face race interacts with facial emotion in an emotion categorization task. In contrast, we predicted the absence of these interactions in Experiments 2 and 3, since the tasks (gender or race categorization) in these experiments demanded a fast processing of invariant facial cues (such as gender or race) rather than a slow processing of variant facial cues (such as emotion).

## Experiment 1: Emotion Categorization Task

### Method

#### Participants

In all four experiments Chinese students, who reported to have normal or corrected-to-normal vision and that they had never lived outside China, participated in exchange of RMB $20 (about 3.5 US dollars). They all gave informed consent at the beginning of the study and were unaware of the purpose of the study. None of them participated in more than one experiment. Twenty (mean age = 21.65 years, *SD* = 1.38 years; three males) students participated in Experiment 1. All experiments reported in the current study were approved by Ethics Committee of Department of Psychology, Renmin University of China.

#### Stimuli and Apparatus

**FIGURE 1:**
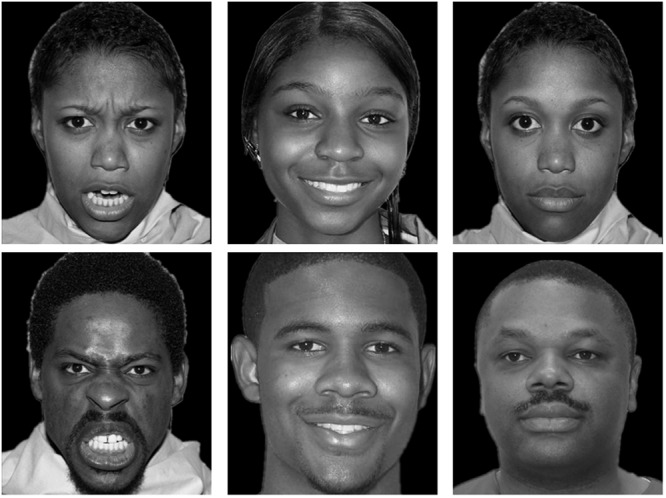
**Examples of Black faces**.

**FIGURE 2:**
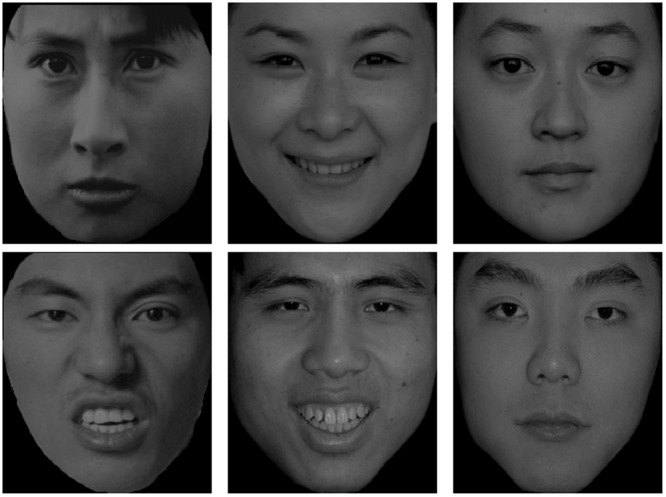
**Examples of Chinese faces**.

**FIGURE 3:**
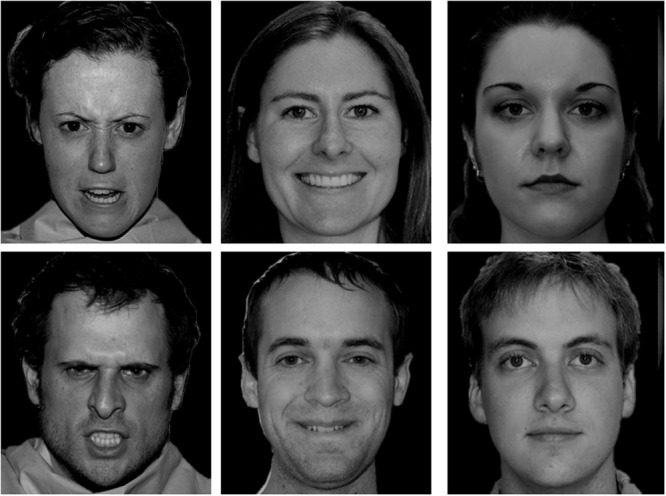
**Examples of Caucasian faces**.

One hundred and eighty black-and-white real face photos of Chinese, White, and Black people were selected from four databases of facial emotion. Half of the photos were female. Those faces wore a happy, angry, or neutral expression. There were 10 male and 10 female face photos for each combination of face race and facial emotion. Chinese faces were chosen from the Chinese Facial Affective Picture System (Gong et al., 2011). White and Black faces were chosen from the Vital Longevity’s Face Database (Minear and Park, 2004), the NimStim Set of Facial Expressions (Tottenham et al., 2009), and the UC Davis Set of Emotion Expression (Tracy et al., 2009). A plus sign (“+”), subtending 1.2° (0.5 cm), served as the fixation point. Each face subtended 6.23° (13.50 cm) in height and 5.39° (12.5 cm) in width. The stimulus presentation and data collection were controlled by E-prime 1.0. All stimuli were presented on a black background. The experiment was run on a Lenovo PC with a 19-inch monitor set to a screen resolution of 1250 × 800 pixels. Another 18 photos drawn from the same set of face databases were used in the practice block. **Figures 1**–**3** showed examples of faces used in the study.

One could argue that selecting face stimuli from different databases might be problematic. For example, individuals may not recognize facial emotions equally well for own and other races (Yankouskaya et al., 2014). Hence, we recruited additional 23 students from the same participant pool as in Experiments 1–3 to rate all faces on emotional valence on a 9-point Likert scale, with 1 = “unpleasant” and 9 = “pleasant”. A 3 (Face race: Chinese, White, or Black) × 3 (Facial emotion: happy, angry, or neutral) × 2 (Face gender: male or female) repeated-measures analysis of variance (ANOVA) was conducted for these ratings. The results showed a main effect of race, *F*(2,44) = 4.57, *p* = 0.027, $\eta_{p}^{2}$ = 0.17, indicating that the mean rating for Chinese faces (*M* = 4.35, *SD* = 0.13) was lower than for Caucasian (*M* = 4.67, *SD* = 0.11) and Black faces (*M* = 4.60, *SD* = 0.12), with no difference in ratings for Caucasian and Black faces. The main effect of emotion was significant, *F*(2,44) = 242.34, *p* < 0.001, $\eta_{p}^{2}$ = 0.91, which showed that happy faces (*M* = 6.92, *SD* = 0.16) were rated more pleasant than neutral (*M* = 4.28, *SD* = 0.15) and angry faces (*M* = 2.43, *SD* = 0.15) and angry faces were rated more unpleasant than neutral faces. The main effect of face gender was also significant, *F*(1,22) = 43.99, *p* < 0.001, $\eta_{p}^{2}$ = 0.66, with lower rating for male faces (*M* = 4.39, *SD* = 0.10) than female faces (*M* = 4.69, *SD* = 0.11). The main effects were qualified by significant face race × facial emotion interaction [*F*(4,88) = 14.77, *p* < 0.001, $\eta_{p}^{2}$ = 0.40] and facial emotion × face gender interaction [*F*(2,44) = 4.58, *p* = 0.029, $\eta_{p}^{2}$ = 0.17]. Simple effect analyses of face race × facial emotion interaction indicated that participants were able to differentiate three types of facial emotions of each race. The mean ratings for Chinese happy, angry, and neutral faces were 6.51 (*SD* = 0.19), 2.63 (*SD* = 0.09), and 3.92 (*SD* = 0.17) [*F*(2,44) = 268.88, *p* < 0.001, $\eta_{p}^{2}$ = 0.92], respectively; for Caucasian faces were 7.12 (*SD* = 0.16), 2.32 (*SD* = 0.21), and 4.58 (*SD* = 0.16) [*F*(2,44) = 200.52, *p* < 0.001, $\eta_{p}^{2}$ = 0.90], respectively; for Black faces were 7.13 (*SD* = 0.18), 2.32 (*SD* = 0.21), and 4.35 (*SD* = 0.17) [*F*(2,44) = 180.38, *p* < 0.001, $\eta_{p}^{2}$ = 0.89], respectively. Simple effect analyses of facial emotion × face gender interaction showed that participants were able to differentiate three types of facial emotions of male and female faces. The mean ratings for male happy, angry, and neutral faces were 6.70 (*SD* = 0.17), 2.33 (*SD* = 0.14), and 4.16 (*SD* = 0.14) [*F*(2,44) = 214.61, *p* < 0.001, $\eta_{p}^{2}$ = 0.90], respectively; for female faces were 7.14 (*SD* = 0.15), 2.52 (*SD* = 0.16), and 4.41 (*SD* = 0.17) [*F*(2,44) = 249.27, *p* < 0.001, $\eta_{p}^{2}$ = 0.92], respectively. These data showed that participants could discriminate three types of facial emotions, although the ratings of emotional faces were modulated by face race and gender. Given that there is no emotional face database for other-race faces in China and other studies used faces drawn from different face databases (e.g., Karnadewi and Lipp, 2011; Craig et al., 2012), we included face stimuli from different databases.

#### Procedure

In each trial, a central fixation appeared for 1000 ms and was then replaced by the picture of a face that remained on the screen for 200 ms (e.g., in Karnadewi and Lipp, 2011). After 200 ms had elapsed, a gray rectangle with the same size as a face picture appeared and remained on the screen until a response was detected. Participants performed an emotion categorization task in which they pressed one of the three keys (“C,” “N,” or “,”) to respond to a happy, angry, or neutral face, respectively. This response key assignment was counterbalanced between participants. There were 360 trials in total, including 10 faces in each of the combinations of face race (Chinese, White, or Black), stimulus repetition (twice), face gender (male or female), and facial emotion (happy, angry, or neutral). Faces of all conditions were presented in a randomized order. Participants were given a practice block of 36 trials at the start of the experiment. There were two self-paced breaks in the experiment.

#### Data Analyses

Reaction time (RT) analyses were conducted for correct trials only. We computed means for each participant in each condition. RTs faster than 200 ms or slower than 3 *SD*s than the mean of each participant in each condition were excluded from the analyses. These trimming criteria discarded 8.2% of the data (5.4% for error responses and 2.8% for extreme responses). We then conducted 3 (Face race: Chinese, White, or Black) × 3 (Facial emotion: happy, angry, or neutral) × 2 (Face gender: male or female) repeated-measures ANOVAs for mean RTs and accuracy rates.

### Results

**Table 1** shows the mean RTs in all conditions. The ANOVA showed three significant main effects: face race [*F*(2,38) = 16.13, *p* < 0.001, $\eta_{p}^{2}$ = 0.45], facial emotion [*F*(2,38) = 13.90, *p* < 0.001, $\eta_{p}^{2}$ = 0.42], and face gender [*F*(1,19) = 5.44, *p* = 0.031, $\eta_{p}^{2}$ = 0.22]. Those main effects were qualified by significant face race × facial emotion interaction, *F*(4,76) = 16.20, *p* < 0.001, $\eta_{p}^{2}$ = 0.46, facial emotion × face gender interaction, *F*(2,38) = 11.19, *p* < 0.001, $\eta_{p}^{2}$ = 0.37, and the three-way interaction, *F*(4,76) = 3.99, *p* = 0.015, $\eta_{p}^{2}$ = 0.17. The face race × gender interaction failed to reach the significance level, *F*(2,38) = 0.65, *p* = 0.51, $\eta_{p}^{2}$ = 0.03.

**Table 1:** Mean RT and accuracy in emotion categorization task (Experiment 1).

	Happy face		Angry face		Neutral face	
	Male	Female	Male	Female	Male	Female
**RT**						
Chinese	558 (152)	514 (131)	636 (188)	681 (221)	609 (182)	599 (195)
White	531 (124)	481 (125)	528 (180)	550 (153)	632 (188)	595 (149)
Black	524 (118)	501 (136)	597 (191)	564 (173)	619 (154)	645 (170)
**Accuracy**						
Chinese	0.94 (0.08)	0.95 (0.05)	0.83 (0.10)	0.84 (0.12)	0.95 (0.06)	0.93 (0.07)
White	0.94 (0.04)	0.97 (0.02)	0.96 (0.04)	0.95 (0.06)	0.88 (0.06)	0.94 (0.05)
Black	0.95 (0.06)	0.96 (0.06)	0.90 (0.08)	0.98 (0.02)	0.93 (0.05)	0.91 (0.06)

*Standard deviations are in parentheses.*

Many studies of face categorization use either only male faces (e.g., Craig et al., 2012) or both male and female faces (e.g., Zhao and Bentin, 2008). Although both male and female faces were included, face gender as a variable has not been well-studied in previous research. It is also not clear whether or not female faces are processed in the same manner as male faces. Hence, the three-way interaction was further analyzed with separate face race × facial emotion repeated-measures ANOVAs for male and female faces. For male faces, the main effects of face race [*F*(2,38) = 7.36, *p* = 0.005, $\eta_{p}^{2}$ = 0.28] and facial emotion [*F*(2,38) = 8.91, *p* = 0.002, $\eta_{p}^{2}$ = 0.32] were significant. There was also a significant face race × facial emotion interaction, *F*(4,76) = 6.63, *p* = 0.001, $\eta_{p}^{2}$ = 0.26. Similarly, for female faces, there were significant main effects of face race [*F*(2,38) = 8.52, *p* = 0.001, $\eta_{p}^{2}$ = 0.31] and facial emotion [*F*(2,38) = 17.96, *p* < 0.001, $\eta_{p}^{2}$ = 0.48]. The face race × facial emotion interaction was also significant, *F*(4,76) = 14.58, *p* < 0.001, $\eta_{p}^{2}$ = 0.43. Thus, female and male faces showed same pattern of results with a difference in effect magnitude.

**Table 1** shows the cell means of accuracy rates in all conditions. An ANOVA for accuracy rates showed a main effect of face race [*F*(2,38) = 11.42, *p* = 0.001, $\eta_{p}^{2}$ = 0.37], a main effect of facial emotion [*F*(2,38) = 8.88, *p* = 0.001, $\eta_{p}^{2}$ = 0.32], and a main effect of face gender [*F*(1,19) = 10.30, *p* = 0.005, $\eta_{p}^{2}$ = 0.32]. These main effects were qualified by significant face race × facial emotion interaction, *F*(4,76) = 17.57, *p* < 0.001, $\eta_{p}^{2}$ = 0.48, and the three-way interaction, *F*(4,76) = 4.20, *p* = 0.009, $\eta_{p}^{2}$ = 0.18. The facial emotion × face gender interaction was not significant, *F*(2,38) = 0.54, *p* = 0.56, $\eta_{p}^{2}$ = 0.33, and the face race × gender interaction failed to reach significance level, *F*(2,38) = 2.38, *p* = 0.10, $\eta_{p}^{2}$ = 0.11. The analyses of accuracy rates shed light on whether there was a speed-accuracy tradeoff in our results. If there is a significant positive effect in RT yet a significant negative effect in accuracy, or vice verse, then it can be concluded that a speed-accuracy tradeoff occurs. On the other hand, if significant effects in RT but merely null effects in accuracy are found, then it does not entail the speed-accuracy tradeoff. Our findings of accuracy rates confirmed that there was no speed-accuracy tradeoff. No further analyses were conducted.

### Discussion

In an emotion categorization task, we obtained significant face race × facial emotion and facial emotion × face gender interactions. The important finding is the significant three-way interaction. According to follow-up analyses, for Chinese and Black male faces, RTs for happy faces were faster than for angry and neutral faces, with no difference between the latter two. For Caucasian male faces, RTs for both happy and angry faces were faster than for neutral faces, with no difference between the happy and angry faces. The face race × facial emotion interaction for female faces showed a slightly different pattern. For Chinese female faces, happy faces were responded to faster than neutral faces, which, in turn, were responded faster than angry faces. However, for Caucasian and Black female faces, happy faces were responded to faster than angry faces, which, in turn, were responded faster than neutral faces. The three-way interaction indicates that when categorizing emotional expressions of Chinese, White, and Black faces as happy, angry, and neutral, participants’ response was affected by facial emotion, face race, and face gender. In other words, the three factors can be simultaneously processed in an emotion categorization task. These results replicated Karnadewi and Lipp’s (2011) finding of the interference of invariant facial cues (race and gender) with the processing of facial emotion, even though participants performed a task that involved the variation of more than one irrelevant invariant facial cue.

## Experiment 2: Race Categorization Task

Previous research (e.g., Kubota and Ito, 2007; Karnadewi and Lipp, 2011) reported a face race × facial emotion interaction in an emotion categorization task, but not in a race categorization task. In Experiment 2, we tested whether this result could be replicated by using a race categorization task with the same set of stimuli as Experiment 1. Participants pressed one of three keys to respond to faces from three races (Chinese, White, and Black). In other words, the task demand (three-alternative-forced-choice) was comparable in this race categorization task with the emotion categorization task in Experiment 1.

### Method

#### Participants

Twenty (mean age = 20.95 years, *SD* = 1.43 years; two males) students participated in this experiment.

#### Stimulus and Apparatus

All were identical to those used in Experiment 1.

#### Procedure

All were the same as those in Experiment 1, except that participants pressed “C,” “N,” or “,” key for a Chinese, White, or Black face.

#### Data Analyses

Data trimming criteria were the same as in Experiment 1. In total, 10.7% of the trials were discarded due to error (8.2%) and extreme responses (2.5%). Data analytic procedures were the same as those in Experiment 1.

### Results

**Table 2** shows the mean RTs in all conditions. There were significant main effects of face race [*F*(2,38) = 29.46, *p* < 0.001, $\eta_{p}^{2}$ = 0.60] and facial emotion [*F*(2,38) = 5.59, *p* = 0.009, $\eta_{p}^{2}$ = 0.22]. The main effect of face gender was not significant, *F*(1,19) = 0.76, *p* = 0.78, $\eta_{p}^{2}$ = 0.004. More importantly, the face race × facial emotion interaction [*F*(4,76) = 8.63, *p* < 0.001, $\eta_{p}^{2}$ = 0.31] and face race × face gender interaction [*F*(2,38) = 18.42, *p* < 0.001, $\eta_{p}^{2}$ = 0.49] were also significant. The facial emotion × face gender interaction [*F*(2,38) = 2.42, *p* = 0.112, $\eta_{p}^{2}$ = 0.11] and the three-way interaction [*F*(4,76) = 2.06, *p* = 0.112, $\eta_{p}^{2}$ = 0.09] failed to reach the significance level.

**Table 2:** Mean RT and accuracy in race categorization task (Experiment 2).

	Happy face		Angry face		Neutral face	
	Male	Female	Male	Female	Male	Female
**RT**						
Chinese	438 (75)	430 (89)	438 (85)	425 (64)	414 (120)	415 (75)
White	557 (128)	498 (102)	568 (108)	557 (150)	515 (101)	499 (104)
Black	451 (123)	483 (90)	441 (128)	507 (122)	470 (126)	490 (103)
**Accuracy**						
Chinese	0.95 (0.05)	0.95 (0.05)	0.93 (0.05)	0.97 (0.03)	0.95 (0.04)	0.96 (0.03)
White	0.87 (0.07)	0.95 (0.05)	0.85 (0.13)	0.94 (0.05)	0.94 (0.05)	0.96 (0.05)
Black	0.93 (0.07)	0.88 (0.09)	0.93 (0.08)	0.88 (0.10)	0.95 (0.06)	0.83 (0.15)

*Standard deviations are in parentheses.*

**Table 2** shows the cell means of accuracy rates in all conditions. An ANOVA for accuracy rates yielded a main effect of face race, *F*(2,38) = 9.50, *p* = 0.003, $\eta_{p}^{2}$ = 0.33. The main effect of facial emotion [*F*(2,38) = 1.93, *p* = 0.16, $\eta_{p}^{2}$ = 0.09] and the main effect of face gender [*F*(1,19) = 0.087, *p* = 0.77, $\eta_{p}^{2}$ = 0.005] were not significant. However, all three two-way interactions reached the significance level: face race × facial emotion, *F*(4,76) = 3.71, *p* = 0.024, $\eta_{p}^{2}$ = 0.16; face race × face gender, *F*(2,38) = 20.21, *p* < 0.001, *F*(1,19) = 10.30, *p* = 0.005, $\eta_{p}^{2}$ = 0.51; facial emotion × face gender, *F*(2,38) = 6.51, *p* = 0.005, *F*(1,19) = 10.30, *p* = 0.005, $\eta_{p}^{2}$ = 0.25. The three-way interaction was not significant, *F*(4,76) = 0.94, *p* = 0.42, $\eta_{p}^{2}$ = 0.04. The results of accuracy rates suggested that there was no speed-accuracy tradeoff.

### Discussion

Experiment 2 adopted a race categorization task that required participants to press one of the three keys to classify Chinese, Caucasian, and Black faces. The results revealed significant face race × facial emotion and face race × face gender interactions. Based on further analyses of face race × facial emotion interaction, RTs for happy and angry faces were slower than for neutral faces in Chinese faces; angry faces were responded to slower than happy faces which, in turn, were responded to slower than neutral faces in Caucasian faces; RTs did not differ for happy, angry, and neutral faces in Black faces. Similarly, the follow-up analyses of face race × face gender interaction showed no gender difference in Chinese faces, faster response to female than to male faces in Caucasian faces, and faster response to male than to female faces in Black faces. The results were not consistent with the absence of the face race × facial emotion interaction in race categorization in some previous studies (e.g., Kubota and Ito, 2007). There were several methodological differences between the present experiment and Kubota and Ito (2007). For example, face stimuli were different (Kubota and Ito, 2007, created their stimuli by taking photos of college students and asking pilot participants to rate those photos, whereas, we selected face photos from face databases). The number of race differed (Kubota and Ito, 2007, included two races, White and Black faces; whereas, we used three races, Chinese, White, and Black faces). The presentation duration of face stimuli was different [750 ms in Kubota and Ito’s (2007) experiment and 200 ms in ours]. In addition, Kubota and Ito (2007) did an ERP (event-related potential) experiment with a half of female and a half of male participants, but the current study was behavioral experiments. At least one of these factors might have contributed to the different results between the current experiment and Kubota and Ito (2007).

## Experiment 3: Gender Categorization Task

A significant three-way interaction was found in both emotion and race categorization tasks. Participants in the first two experiments performed a ternary-response task, so some could argue that the interactions of different facial properties might only be observed in a demanding task. In Experiment 3, we adopted a binary-response gender categorization task to further explore the interactions of these factors.

### Method

#### Participants

Twenty-two students (mean age = 21.68 years, *SD* = 3.09 years; three males) participated in Experiment 3.

#### Stimuli and Apparatus

All were identical to those of Experiment 1.

#### Procedure

All were identical to those of Experiment 1, except the task demand and response key assignment. Participants pressed either “C” or “M” key to respond to a male or a female face. The key assignment was counterbalanced between participants.

#### Data Analyses

Data trimming and data analytic procedures were the same as those in Experiment 1. There were 12.2% of trials that were discarded (9.6% for error responses and 2.6% for extreme responses).

### Results

**Table 3** shows the mean RTs in all conditions. There were significant main effects of face race [*F*(2,42) = 55.04, *p* < 0.001, $\eta_{p}^{2}$ = 0.72] and facial emotion [*F*(2,42) = 8.12, *p* = 0.002, $\eta_{p}^{2}$ = 0.28]. The main effect of face gender failed to reach the significance level, *F*(1,21) = 1.26, *p* = 0.274, $\eta_{p}^{2}$ = 0.05. The face race × facial emotion [*F*(4,84) = 4.10, *p* = 0.037, $\eta_{p}^{2}$ = 0.16], face race × face gender [*F*(2,42) = 15.45, *p* < 0.001, $\eta_{p}^{2}$ = 0.42], and facial emotion × face gender interactions [*F*(2,42) = 26.10, *p* < 0.001, $\eta_{p}^{2}$ = 0.55] were all significant. The three-way interaction was not significant, *F*(4,84) = 2.58, *p* = 0.075, $\eta_{p}^{2}$ = 0.11.

**Table 3:** Mean RT and accuracy in gender categorization task (Experiment 3).

	Happy face		Angry face		Neutral face	
	Male	Female	Male	Female	Male	Female
**RT**						
Chinese	432 (134)	382 (122)	437 (192)	460 (122)	436 (127)	406 (109)
White	378 (105)	344 (85)	364 (99)	365 (93)	403 (142)	356 (116)
Black	352 (133)	376 (120)	354 (97)	395 (89)	354 (98)	357 (88)
**Accuracy**						
Chinese	0.86 (0.09)	0.92 (0.10)	0.92 (0.07)	0.61 (0.17)	0.88 (0.08)	0.89 (0.11)
White	0.93 (0.07)	0.97 (0.03)	0.97 (0.04)	0.93 (0.07)	0.93 (0.05)	0.97 (0.04)
Black	0.96 (0.04)	0.90 (0.06)	0.95 (0.05)	0.86 (0.12)	0.95 (0.06)	0.95 (0.05)

*Standard deviations are in parentheses.*

**Table 3** shows the cell means of accuracy rates in all conditions. The analyses of accuracy rates yielded significant main effects of face race [*F*(2,42) = 47.49, *p* < 0.001, $\eta_{p}^{2}$ = 0.69], facial emotion [*F*(2,42) = 30.53, *p* < 0.001, $\eta_{p}^{2}$ = 0.59], and face gender [*F*(1,21) = 11.31, *p* = 0.003, $\eta_{p}^{2}$ = 0.35]. All two-way and three-way interactions were also significant: face race × facial emotion [*F*(4,84) = 13.33, *p* < 0.001, $\eta_{p}^{2}$ = 0.38]; face race × face gender [*F*(2,42) = 8.54, *p* = 0.001, $\eta_{p}^{2}$ = 0.29); facial emotion × face gender [*F*(2,42) = 72.57, $\eta_{p}^{2}$ = 0.77]; and the three-way interaction [*F*(4,84) = 24.89, *p* < 0.001, $\eta_{p}^{2}$ = 0.54]. No further analyses were conducted on accuracies.

### Discussion

In the gender categorization task, all the two-way interactions were significant, in contrast to that Experiments 1 and 2 failed to reveal some two-way interactions of task-irrelevant features (face race and gender in Experiment 1; facial emotion and face gender in Experiment 2). According to further analyses of face race × facial emotion interaction, Chinese happy and angry faces were responded to faster than Chinese neutral faces. No difference in RTs was observed for Caucasian happy, angry, and neutral faces. RT for Black angry faces was slower than for Black neutral faces, and RT for Black happy faces did not differ from Black angry and neutral faces. The follow-up analyses of face gender × facial emotion interaction revealed no difference in RTs for male happy, angry, and neutral faces and faster RTs for both female happy and angry faces than for female neutral faces. The further analyses of face race × face gender interaction indicated no gender effect for Chinese faces, faster RT for Caucasian female than for male faces, and faster RT for Black male than female faces.

## General Discussion

Many researchers agree that individuals can process more than one feature in face perception, but they debate on whether multiple features are processed independently or simultaneously. For example, Kubota and Ito (2007) provided evidence for independent processing of face race and facial emotion in an emotion categorization task. However, other works revealed interactive processing of face race and facial emotion (e.g., Hugenberg, 2005; Zebrowitz et al., 2010). The inconsistent results were also found in the processing of face race and face gender, facial emotion and face gender, and so on. Even if for studies that obtained interactive process of two face features, there was a debate on whether or not the interaction is symmetric. Using the same set of stimuli and participant population, the current three experiments tested the independent versus simultaneous processing of three face features (race, gender, and facial emotion) in various categorization tasks.

The most important findings of the current study are the significant facial emotion × face race interaction in emotion and race categorization tasks, the facial emotion × face gender interaction in emotion and gender categorization tasks, and the face race × face gender interaction in race and gender categorization tasks. These critical findings showed that interactions between variant (e.g., facial emotion) and invariant face features (e.g., race and gender) were symmetrical. According to Cohen (1988), an effect with partial eta square equal to or larger than 0.14 is considered a large effect. The effect sizes were all larger than 0.30 in the above interaction effects across our three experiments, suggesting that the effects, we obtained were large according to Cohen’s criteria. The symmetric interactions of invariant and variant face features and of invariant face features suggests that face race, face gender, and facial emotion are processed simultaneously and in turn influence participants’ decisions in emotion, race, and gender categorization tasks. Previous studies also showed that facial emotion could be simultaneously processed with identity and race, at least in individuals with more exposure to other ethnicities (Yankouskaya et al., 2014).

In the emotion categorization task, invariant cues such as face race and face gender automatically influenced the processing of variant cues such as facial emotion. However, in Experiments 2 and 3, variant cues also interacted with the processing of invariant cues. While these results were in line with those reported in some studies (Craig et al., 2014; Experiment 2 on the effect of facial emotion in a race-focused priming task), they were not fully consistent with the results of Karnadewi and Lipp (2011).

The discrepancy between the current study and Karnadewi and Lipp (2011) could be attributed to the following procedural differences. First, three instead of two facial properties were manipulated. The processing of multiple facial cues might be more demanding when the number of cues involved increases. Second, whereas Karnadewi and Lipp (2011) used the Garner paradigm, the current study used binary and three-alternative forced-choice face categorization tasks. Different paradigms or tasks may encourage participants to adopt different response strategies, which results in different patterns of findings. For example, in the Garner paradigm, participants make a binary response in which they could use an A/not-A strategy when responding. Thus, A represents one of the two responses. However, in a three-alternative forced-choice categorization task, participants make a ternary response in which the A/not-A strategy is no longer helpful. Finally, unlike the present study, Karnadewi and Lipp (2011) did not include neutral faces. A neutral condition is necessary in a study on emotion effect because different emotions, such as happiness and anger, might influence the dependent variable in a similar manner. In Experiment 2, participants’ overall responses were similar to happy and angry faces, but their responses to happy and angry faces were both faster than neutral faces. If no neutral face were included, a null effect of emotion on race categorization would have been obtained.

Other studies directly investigated the factors that contribute to the inconsistency of the findings of multiple face features processing. Craig et al. (2012) pointed out that stimulus type, set size, and duration had effects on face race × facial emotion interaction. Lipp et al. (2015) also suggested that stimulus set size influenced the face gender × facial emotion interaction. The current study used real face pictures and large stimulus set size and found symmetrical face race × facial emotion and face gender × facial emotion interactions, which was consistent with previous findings (Craig et al., 2012; Lipp et al., 2015). Further research should systematically manipulate these parameters in order to test whether our current findings would be generalized in other circumstances.

In each of the categorization tasks, while one face feature is task-relevant, the other two features are task-irrelevant. Previous research on processing of face features showed that some face features such as facial emotion, face race, face gender could capture attention and be processed automatically on a early stage even though those features are task-irrelevant (Mouchetant-Rostaing et al., 2000; Vuilleumier et al., 2001; Ito and Urland, 2003, 2005; Hodsoll et al., 2011; Ambron and Foroni, 2015). The current finding supported the automatic processing of facial emotion, face race, and face gender, because the task-irrelevant features interact with task-relevant feature in both emotion and race categorization task and two task-irrelevant features interact with one another in the gender categorization task. Both task-relevant and task-irrelevant features are processed simultaneously in the three categorization tasks, which is consistent with Ito and Urland’s (2005) idea on the simultaneous processing of face features.

Researchers have debated on the theoretical mechanism of face processing. For example, Bruce and Young (1986) proposed that structural encoding and social categorization were distinct stages, with structural encoding prior to social categorization. However, Ito and Urland (2005) argued that social categorization processing did not need to depend on structural encoding because social categorization occurred very early (before 170 ms) based on some ERP studies on face race and gender (e.g., Mouchetant-Rostaing et al., 2000; Ito and Urland, 2003, 2005; Mouchetant-Rostaing and Giard, 2003). In response to this, Ito and Urland (2005) proposed a model depicting simultaneous, rather than sequential, processing of structural encoding and social categorization.

According to the Haxby et al. (2000) face-processing model, separate neural systems are responsible for analyzing the variant and invariant facial cues. The automatic processing of one invariant cue affects the analysis of another invariant cue (Zhao and Hayward, 2013). Given that both face race and face gender are invariant facial cues, Haxby et al. (2000) model could predict a face race × face gender interaction for each of the current experiments. However, this interaction was not consistently obtained in the current study. No significant face race × face gender interaction was found in the emotion categorization task. Also, Haxby et al. (2000) model indicates different processes of invariant and variant facial cues, such as the faster processing of race and slower processing of facial emotions. If invariant cues affected the processing of variant cues but not vice versa (due to differential speed in the processing of these two facial cues), then there would have only been a facial emotion × face race interaction and a facial emotion × face gender interaction in the emotion categorization task. The present results indicated that the processing of multiple facial cues might be more complex than previously described in Haxby et al. (2000) model.

Craig et al. (2014) argue that an interactive model of face perception, such as the Dynamic Interactive Theory of Person Construal (Freeman and Ambady, 2011), is better than the separate processing model in explaining the processing of multiple facial cues. The interactive model highlights the interaction between top-down (goals, task demands) and bottom-up influences in face perception. In the emotion, race, and gender categorization tasks, task demands varied across experiments, which lead to different processing priorities relevant to the facial cues. For example, in the gender categorization task, gender cues were task-relevant and became the most salient cues. However, the processing of gender cues was also affected by some bottom-up activation of emotion and race cues. Depending on which cues were salient, the nature of interaction of those cues changed accordingly.

One limitation of the current study is that majority of the participants are females, who may have been more sensitive to emotional and social cues (Bayliss and Tipper, 2005; Deaner et al., 2007). The symmetric interaction between variant and invariant facial cues might be specific for female participants. Nevertheless, evidence has been mixed on whether participant’s gender could have an effect on gender categorization. On one hand, O’Toole et al. (1996) found female participants were more accurate in the gender categorization task than male participants. On the other hand, Zhao and Bentin (2008) failed to observe any difference in gender categorization task between female and male participants. We re-did our analyses only on female participants’ data (see the Appendix). Critically, the three-way interactions in race and gender categorization tasks became significant even in RTs. This might suggest that participant’s gender might influence the higher-order interaction. Future research should use a more balanced number of male and female participants to explore this issue.

Another potential limitation is that faces used in the current study are selected from different face databases. Although other studies also include faces from different databases (e.g., Karnadewi and Lipp, 2011; Craig et al., 2012), there are some potential problems worth to be mentioned. For example, Chinese faces are more closely cropped than the Caucasian and Black faces that have more external feature like hair. These differences in lower-level features may have an effect on RTs. However, previous findings indicated that lower-level features did Ito and Urland, 2003). The lower-level face features might serve as confounding variables, but they cannot explain our symmetrical interactions. A better control over the face stimuli should be made in the future studies.

## Conclusion

The current results support the hypothesis that facial emotion, face race and face gender could be simultaneously processed, even when these three attributes were varied and some of the attributes were task irrelevant. Variant and invariant facial cues interfered with one another; and the interaction was symmetric. As such, automatically processed facial properties may result in symmetric interference in certain conditions. However, the way in which the two types of cues interact may be different for different tasks. The present findings provide evidence for the face processing models that support the interactive processing of multiple facial cues.

## Author Contributions

YL designed the experiments, collected and analyzed data, wrote the first draft of the manuscript. C-ST made revisons of the manuscript.

## Conflict of Interest Statement

The authors declare that the research was conducted in the absence of any commercial or financial relationships that could be construed as a potential conflict of interest.

## Appendix

### Results for Experiments 1–3 with Female Participants Only

#### Participants

Seventeen (mean age = 21.64 years, *SD* = 1.45 years), eighteen (mean age = 20.88 years, *SD* = 1.49 years), and nineteen (mean age = 21.84 years, *SD* = 3.14 years) students participated in Experiments 1, 2, and 3, respectively.

#### Data Analyses

Reaction time (RT) analyses were conducted for correct trials only. We computed means for each participant in each condition. RTs faster than 200 ms or slower than 3 *SD*s than the mean of each participant in each condition were excluded from the analyses. These trimming criteria discarded 3.6% of the data in Experiment 1, 6.9% in Experiment 2, and 7.2% in Experiment 3.

### Results for Experiment 1: Emotion Categorization Task

There were significant main effects of face race [*F*(2,32) = 12.76, *p* < 0.001, $\eta_{p}^{2}$ = 0.44] and facial emotion [*F*(2,32) = 16.54, *p* < 0.001, $\eta_{p}^{2}$ = 0.50]. The main effect of face gender was not significant, *F*(1,16) = 3.73, *p* = 0.07, $\eta_{p}^{2}$ = 0.18. Those main effects were qualified by a significant face race × facial emotion interaction [*F*(4,64) = 14.34, *p* < 0.001, $\eta_{p}^{2}$ = 0.47] and a significant facial emotion × face gender interaction [*F*(2,32) = 8.66, *p* = 0.001, $\eta_{p}^{2}$ = 0.35]. Most importantly, the three-way interaction of face race, facial emotion, and face gender reached the significance level, *F*(4,64) = 3.50, *p* = 0.03, $\eta_{p}^{2}$ = 0.18. However, the face race × face gender interaction failed to reach the significance level, *F*(2,32) = 0.59, *p* = 0.54, $\eta_{p}^{2}$ = 0.03.

The three-way interaction was further analyzed with separate face race × facial emotion repeated-measures ANOVAs for male and female faces. For male faces, the main effects of face race [*F*(2,32) = 5.75, *p* = 0.01, $\eta_{p}^{2}$ = 0.26] and facial emotion [*F*(2,32) = 11.56, *p* = 0.001, $\eta_{p}^{2}$ = 0.42] were significant. There was also a significant face race × facial emotion interaction, *F*(4,64) = 5.33, *p* = 0.003, $\eta_{p}^{2}$ = 0.25. Similarly, for female faces, there were significant main effects of face race [*F*(2,32) = 6.68, *p* = 0.004, $\eta_{p}^{2}$ = 0.29] and facial emotion [*F*(2,32) = 19.42, *p* < 0.001, $\eta_{p}^{2}$ = 0.54], and the face race × facial emotion interaction was significant, *F*(4,64) = 13.47, *p* < 0.001, $\eta_{p}^{2}$ = 0.45.

An ANOVA for accuracy rates showed a main effect of face race [*F*(2,32) = 7.87, *p* = 0.005, $\eta_{p}^{2}$ = 0.33], a main effect of facial emotion [*F*(2,32) = 8.10, *p* = 0.002, $\eta_{p}^{2}$ = 0.33], and a main effect of face gender [*F*(1,16) = 7.78, *p* = 0.013, $\eta_{p}^{2}$ = 0.32]. The face race × facial emotion interaction was significant, *F*(4,64) = 16.20, *p* < 0.001, $\eta_{p}^{2}$ = 0.50. Also, the three-way interaction was significant, *F*(4,64) = 2.84, *p* = 0.04, $\eta_{p}^{2}$ = 0.15. Other interactions failed to reach the significance level, face race × face gender [*F*(2,32) = 1.34, *p* = 0.27, $\eta_{p}^{2}$ = 0.07], facial emotion × face gender [*F*(2,32) = 0.05, *p* = 0.94, $\eta_{p}^{2}$ = 0.003].

### Results for Experiment 2: Race Categorization Task

There were significant main effects of face race [*F*(2,34) = 24.34, *p* < 0.001, $\eta_{p}^{2}$ = 0.58] and facial emotion [*F*(2,34) = 6.70, *p* = 0.004, $\eta_{p}^{2}$ = 0.28]. The main effect of face gender was not significant, *F*(1,17) = 0.21, *p* = 0.64, $\eta_{p}^{2}$ = 0.01 The two main effects were moderated by a significant face race × facial emotion interaction [*F*(4,68) = 8.25, *p* < 0.001, $\eta_{p}^{2}$ = 0.32] and a face race × face gender interaction [*F*(2,34) = 26.80, *p* < 0.001, $\eta_{p}^{2}$ = 0.61]. The three-way interaction was also significant, *F*(4,68) = 2.78, *p* = 0.05, $\eta_{p}^{2}$ = 0.14. The facial emotion × face gender interaction failed to reach the significance level, *F*(2,34) = 2.10, *p* = 0.15, $\eta_{p}^{2}$ = 0.11.

The three-way interaction was explored with separate face race × facial emotion repeated-measures ANOVAs for male and female faces. For male faces, there was a significant main effect of face race, *F*(2,34) = 25.87, *p* < 0.001, $\eta_{p}^{2}$ = 0.60, and a significant face race × facial emotion interaction, *F*(4,68) = 8.08, *p* < 0.001, $\eta_{p}^{2}$ = 0.32. The main effect of facial emotion was not significant, *F*(2,34) = 3.21, *p* = 0.06, $\eta_{p}^{2}$ = 0.15. For female faces, The main effects of face race [*F*(2,34) = 22.50, *p* < 0.001, $\eta_{p}^{2}$ = 0.57] and facial emotion [*F*(2,34) = 6.48, *p* = 0.005, $\eta_{p}^{2}$ = 0.27] were significant. However, the face race × facial emotion interaction failed to reach significance level, *F*(4,68) = 2.13, *p* = 0.10, $\eta_{p}^{2}$ = 0.11. These results indicate that female and male faces were processed differently in a race categorization task.

An ANOVA for accuracy rates yieldeda main effect of face race, [*F*(2,34) = 8.47, *p* = 0.005, $\eta_{p}^{2}$ = 0.32]. The main effects of facial emotion [*F*(2,34) = 1.32, *p* = 0.27, $\eta_{p}^{2}$ = 0.06] and face gender [*F*(1,17) = 0.003, *p* = 0.95, $\eta_{p}^{2}$ = 0.00] were not significant. Three two-way interactions were significant: face race × facial emotion, *F*(4,68) = 3.75, *p* = 0.02, $\eta_{p}^{2}$ = 0.17; face race × face gender, *F*(2,34) = 20.37, *p* < 0.001, $\eta_{p}^{2}$ = 0.53; facial emotion × face gender, *F*(2,34) = 8.08, *p* = 0.002, $\eta_{p}^{2}$ = 0.31. The three-way interaction was not significant, *F*(4,68) = 1.38, *p* = 0.26, $\eta_{p}^{2}$ = 0.07.

### Results for Experiment 3: Gender Categorization Task

There were significant main effects of face race [*F*(2,36) = 56.62, *p* < 0.001, $\eta_{p}^{2}$ = 0.76] and facial emotion [*F*(2,36) = 11.24, *p* < 0.001, $\eta_{p}^{2}$ = 0.38]. The face race × facial emotion [*F*(4,72) = 6.48, *p* = 0.001, $\eta_{p}^{2}$ = 0.26], face race × face gender [*F*(2,36) = 13.48, *p* < 0.001, $\eta_{p}^{2}$ = 0.42], and facial emotion × face gender [*F*(2,36) = 33.59, *p* < 0.001, $\eta_{p}^{2}$ = 0.65] interactions were all significant. The three-way interaction was also significant, *F*(4,72) = 7.23, *p* < 0.001, $\eta_{p}^{2}$ = 0.28. The main effect of face gender failed to reach the significance level, *F*(1,18) = 0.19, *p* = 0.66, $\eta_{p}^{2}$ = 0.01.

The three-way interaction was explored with separate face race × facial emotion repeated-measures ANOVAs for male and female faces. For male faces, ANOVA revealed a significant main effect of face race, *F*(2,36) = 39.78, *p* < 0.001, $\eta_{p}^{2}$ = 0.68. The main effect of facial emotion [*F*(2,36) = 2.11, *p* = 0.13, $\eta_{p}^{2}$ = 0.10] and the face race × facial emotion interaction [*F*(4,72) = 2.01, *p* = 0.12, $\eta_{p}^{2}$ = 0.10] were not significant. For female faces, there was a significant main effect of face race, *F*(2,36) = 38.47, *p* < 0.001, $\eta_{p}^{2}$ = 0.68 and a significant main effect of facial emotion, *F*(2,36) = 39.77, *p* < 0.001, $\eta_{p}^{2}$ = 0.68. The face race × facial emotion interaction was also significant, *F*(4,72) = 11.42, *p* < 0.001, $\eta_{p}^{2}$ = 0.38. More facial properties of female faces were processed simultaneously as compared with male faces.

The analysis of accuracy rates yielded significant main effects of face race [*F*(2,36) = 51.45, *p* < 0.001, $\eta_{p}^{2}$ = 0.74], face gender [*F*(1,18) = 10.84, *p* = 0.004, $\eta_{p}^{2}$ = 0.37], and facial emotion [*F*(2,36) = 30.09, *p* < 0.001, $\eta_{p}^{2}$ = 0.62]. All two-way and three-way interactions were also significant: face race × facial emotion [*F*(4,72) = 13.67, *p* < 0.001, $\eta_{p}^{2}$ = 0.43]; face race × face gender [*F*(2,36) = 8.38, *p* = 0.002, $\eta_{p}^{2}$ = 0.32]; facial emotion × face gender [*F*(2,36) = 71.46, $\eta_{p}^{2}$ = 0.79]; and the three-way interaction [*F*(4,72) = 25.67, *p* < 0.001, $\eta_{p}^{2}$ = 0.58]. No further analyses were conducted on accuracies.

### Discussion

The results of female participants showed a facial emotion × face race interaction in both emotion and race categorization tasks, a facial emotion × face gender interaction in both emotion and gender categorization tasks, and a face gender × face race interaction in both race and gender categorization tasks. Those two-way interactions were symmetrical. In addition, there was a three-way interaction in all three experiments. The results supported the simultaneous processing of facial emotion, face race, and face gender. The invariant features not only interact with other invariant features, but also interact with variant features. So does the variant features.
